# Functional Characterization of Four Known Cav2.1 Variants Associated with Neurodevelopmental Disorders

**DOI:** 10.3390/membranes13010096

**Published:** 2023-01-11

**Authors:** Mathilde Folacci, Sébastien Estaran, Claudine Ménard, Anaïs Bertaud, Matthieu Rousset, Julien Roussel, Jean-Baptiste Thibaud, Michel Vignes, Alain Chavanieu, Pierre Charnet, Thierry Cens

**Affiliations:** IBMM, Université de Montpellier, CNRS, ENSCM, 1919 Route de Mende, Pôle Chimie Bâlard Recherche, CEDEX 5, 34293 Montpellier, France

**Keywords:** channelopathy, Cav2.1, Ca^2+^ channel, electrophysiology, *Xenopus laevis* oocytes

## Abstract

Cav2.1 channels are expressed throughout the brain and are the predominant Ca^2+^ channels in the Purkinje cells. These cerebellar neurons fire spontaneously, and Cav2.1 channels are involved in the regular pacemaking activity. The loss of precision of the firing pattern of Purkinje cells leads to ataxia, a disorder characterized by poor balance and difficulties in performing coordinated movements. In this study, we aimed at characterizing functional and structural consequences of four variations (p.A405T in I-II loop and p.R1359W, p.R1667W and p.S1799L in IIIS4, IVS4, and IVS6 helices, respectively) identified in patients exhibiting a wide spectrum of disorders including ataxia symptoms. Functional analysis using two major Cav2.1 splice variants (Cav2.1+e47 and Cav2.1−e47) in *Xenopus laevis* oocytes, revealed a lack of effect upon A405T substitution and a significant loss-of-function caused by R1359W, whereas R1667W and S1799L caused both channel gain-of-function and loss-of-function, in a splice variant-dependent manner. Structural analysis revealed the loss of interactions with S1, S2, and S3 helices upon R1359W and R1667W substitutions, but a lack of obvious structural changes with S1799L. Computational modeling suggests that biophysical changes induced by Cav2.1 pathogenic mutations might affect action potential frequency in Purkinje cells.

## 1. Introduction

**Figure 1:**
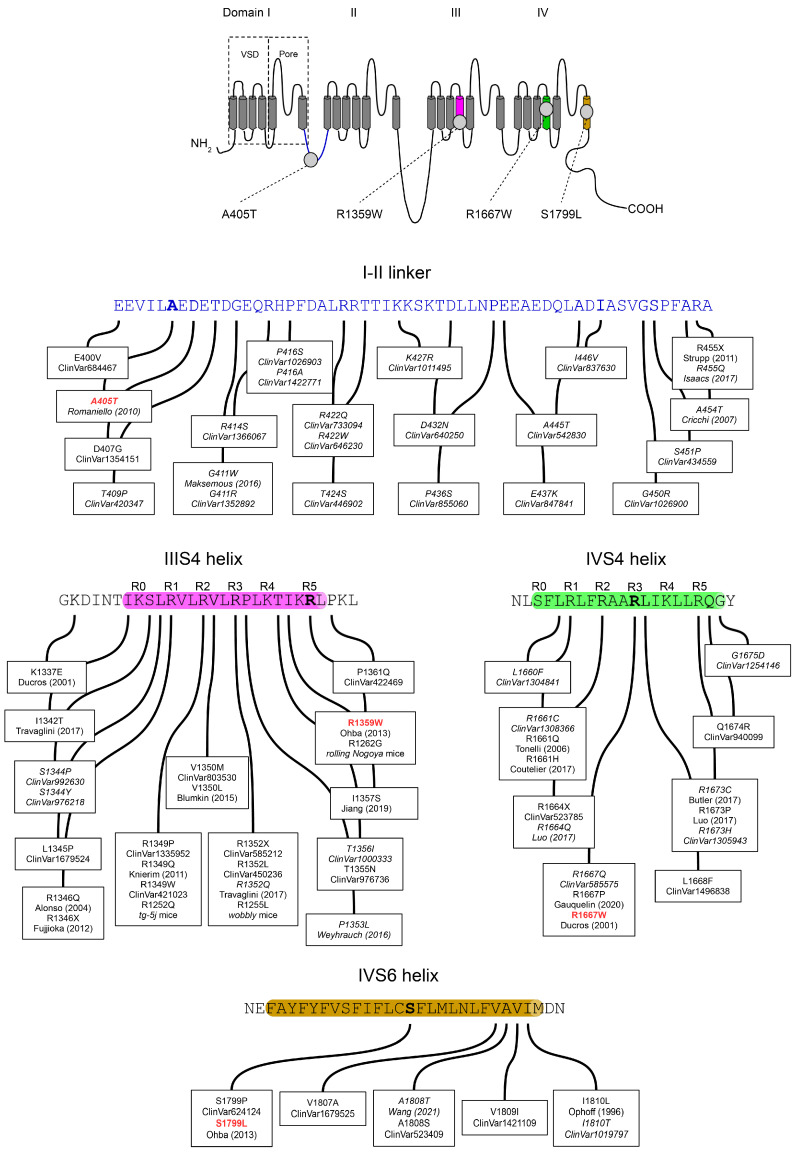
(**Top**) Topology of the voltage-gated Cav2.1 subunit with the position of the four pathogenic variants studied: A405T, R1359W, R1667W, and S1799L. The Cavα is formed of four repeats (I to IV) each containing 6 transmembrane helices (S1–S6). The S1–S4 helices constitute the voltage-sensing domain (VSD) able to move in response to membrane potential changes. The four re-entrant P loops between segments S5 and S6 carry the selectivity filter and delineate the extracellular end of the channel pore. The S5 and S6 helices of the four repeats delineate the cytoplasmic end of the channel pore. (**Bottom**) Amino-acid sequences of the I-II loop, and of the IIIS4, IVS4, and IVS6 helices. Cav2.1 variants referenced in the NCBI ClinVar database are indicated with their accession number. References in *italics* correspond to variants ‘of uncertain significance’ when subjected to in silico analysis. The ‘R’ above the sequences of the IIIS4 and IVS4 helices indicates the position of a basic residue. The Cav2.1 sequence is numbered according to the NCBI Genbank^®^ sequence AAB64179.

By mediating spatially and temporarily regulated Ca^2+^ entry, voltage-gated Ca^2+^ channels participate in many biological processes such as muscle contraction, hormone or neurotransmitter release, and gene expression [1]. The precise Ca^2+^ signaling is achieved thanks to the existence of three families (Cav1–3) of voltage-gated Ca^2+^ channels with distinctive biophysical and pharmacological properties. While Cav3 Ca^2+^ channels (three members Cav3.1 to 3.3) can form functional channels alone, typical Cav1 (four members Cav1.1 to 1.4) and Cav2 (three members Cav2.1 to 2.3) Ca^2+^ channels include with the pore-forming Cavα subunit (Cav1.X or Cav2.X), one of the four Cavβ (Cavβ1 to 4) and one of the four Cavα2δ (Cavα2δ to 4) auxiliary subunits, which modulate properties, expression and regulation of Cavα [2]. The diversity of Ca^2+^ channels resulting from the different Cavα/β/α2δ combinations of subunits can be further accentuated by the alternative splicing of Cavα and/or their auxiliary subunits. For example, alternative splicing produces Cav2.1 subunits with distinct cytoplasmic C-terminus, Cav2.1−e47 and Cav2.1+e47 [3]. The Cavα subunit has 24 transmembrane helices organized into four repeats (I to IV), each with 6 transmembrane helices (S1 to S6) forming two functional domains: the voltage sensing domain (VSD: S1–S4) and the pore domain (S5–S6) (Figure 1). The S4 helices contain several positively charged residues, either R or K, at every third or fourth position (Figure 1), which are known as the gating charges. In fact, S4 helices constitute the actual voltage-sensor able to move across the electric field of the membrane potential [4]. The linker connecting the S5 and S6 helices forms a re-entrant P loop carrying a selectivity filter (the EEEE locus) which allows the discrimination of Ca^2+^ from other ions (see also [5]). The P loops adopt a funnel-like shape with the selectivity filter at their bottom [6,7]. Under the P loops, S5 and S6 helices edge a larger cavity where Ca^2+^ ions flow. The cytoplasmic ends of S6 helices seal the cavity and hydrophobic residues in each S6 delineate the activation gate. At rest, negative membrane potential pulls S4 helices towards the intracellular compartment (S4s are in the ‘down’ position), which maintains the activation gate closed. Upon membrane depolarization, S4 helices are attracted towards extracellular space (S4s are in the ‘up’ position), which induces structural rearrangements in the VSDs leading to the opening of the activation gate and channel activation [8]. The translocation of gating charges across the membrane is facilitated by transient interactions with negative countercharges in S1–S3 helices [9]. During the membrane depolarization, while the S4 helices are still ‘up’, subsequent structural changes cause the closing of the activation gate and channel inactivation. The voltage-dependent inactivation process involves, together with VSD and pore domains, the intracellular loop connecting repeat I and II, and the cytoplasmic amino- and carboxy-terminus [10].

Cav2.1 channels are present throughout the brain and are involved in fast neurotransmitter release at pre-synaptic zones and participate in determining the cellular excitability of the somatodendritic region in post-synaptic neurons [11]. They are prevalent in the cerebellum and particularly in the Purkinje cells, where Cav2.1 subunits are preferentially associated with the Cavβ4 subunit [12]. The critical role played by Cav2.1 is emphasized by the phenotype of Cav2.1 knock-out mice [13], which exhibit ataxia, a disorder characterized by poor balance and loss of posture and movement coordination. There are several spontaneously occurring mouse strains showing mild to severe progressive ataxia in which mutations have been discovered in Cav2.1 (*tottering*, *leaner*, *rolling Nagoya*, *rocker*…), Cavβ4 (*lethargic*), or Cavα2δ (*ducky*) subunits [14]. In Purkinje cells of *ducky*, *leaner*, and *tottering* mice, the precision of the intrinsically driven pacemaking activity is altered, which might be the cause of the ataxic phenotype [15]. Since the end of the 1990s, three autosomal dominant disorders are associated with mutations in *CACNA1A*, the gene that encodes the Cav2.1 subunit: Episodic Ataxia type 2 (EA2, OMIM #108500), Familial Hemiplegic Migraine type 1 (FHM1, OMIM #141500) and Spinocerebellar Ataxia type 6 (SCA6, OMIM #183086) [16]. EA2 is characterized by paroxysmal attacks of ataxia and dysarthria lasting for several hours, with early onset during childhood or adolescence; FHM1 by attacks of migraines accompanied by auras with transient motor weakness of variable severity; and SCA6 by a slowly progressive cerebellar syndrome with adult-onset around 40–50 years [17]. Although EA2, FHM1, and SCA6 are distinct disorders, in some patients, phenotypes overlap and the co-occurrence of distinct phenotypes within individuals of the same family sharing the same *CACNA1A* mutation is not infrequent [18]. Consequently, it has been suggested that EA2, FHM1, and SCA6 might be the same disorder with great phenotype variability [19] (see also [20]). Recently, pathogenic *CACNA1A* variants were identified as an important cause of Epileptic Encephalopathy type 42 (EIEE42, OMIM #617106) characterized by the onset of various seizure types early after birth, global developmental delay and intellectual disability [21]. Finally, mutations in the Cav2.1 subunit have been associated with Congenital Ataxia characterized by chronic cerebellar syndromes with acute symptoms of EA2 or FHM1 [22], and the number of disorders in which *CACNA1A* mutations are involved might continue to increase [20].

Next-generation sequencing approaches (whole-genome, whole-exome, or targeted genes sequencing) are an increasingly practical tool for the molecular diagnosis of neurological disorders [23,24]. ClinVar (https://www.ncbi.nlm.nih.gov/clinvar/ (accessed on 28 October 2022)) includes hundreds of *CACNA1A* variants, among which 309 are categorized as pathogenic or likely pathogenic. Many disease-causing mutations seem to be preferentially located in the S4 and the S5-S6 helices of the different repeats [22,25]. In this study, we aimed to characterize the functional consequences of four *CACNA1A* mutations identified in patients exhibiting a wide spectrum of disorders. The first of these mutations, A405T, is located in the intracellular loop connecting repeat I and II (Figure 1); R1359W and R1667W affect one of the gating charges in IIIS4 and IVS4 helices, respectively; and finally, S1799L is located in the midst of S6 helix in repeat IV. Such analyses are important, not only to understand the pathophysiological mechanism of the diseases but also to shed new light on Ca^2+^ channel structure-function relationships. We found that A405T substitution does not impact Cav2.1 voltage-dependent properties, whereas R1359W causes a channel loss-of-function by stabilizing the inactivated state. On the other hand, R1667W and S1799L mutations cause both channel loss-of-function and gain-of-function, in a splice variant-dependent manner.

## 2. Materials and Methods

### 2.1. Molecular Biology

The cDNAs encoding the Cav2.1 +e47 (NCBI Genbank^®^ accession number AAB64179), Cavβ4a (NP_001386072), and Cavα2δ1 (NP_037061) subunits were cloned into the pcDNA3.1(+), pMT2, and pcDNA3.1(−) expression vectors, respectively. For mutagenesis, Cav2.1 cDNA was cloned into pBluescript II. The R1359W, R1667W, and S1799L mutations were introduced into the Cav2.1 coding sequence by using the NEBuilder^®^ HiFi Assembly kit (NewEngland Biolabs, Ipswich, MA, USA). For each mutant, two pairs of oligonucleotides were designed using the NEbuilder^®^ assembly tool (https://nebuilder.neb.com, accessed on 28 October 2022), and two fragments were PCR amplified using the Herculase II fusion polymerase (Agilent, Santa Clara, CA, USA) and the Cav2.1 cDNA as template. The two fragments and the pBluescript II-Cav2.1+e47 plasmid (opened with AatII and BstEII restriction enzymes) were then assembled following manufacturer instruction. For A405T, a synthetic fragment carrying the desired mutation was purchased from Eurofins Genomics (Ebersberg, Germany). This fragment was used to exchange the original sequence by conventional molecular biology techniques. Mutation and sequence integrity were verified by sequencing the amplified/synthetic fragments on both stands. Each cDNA (Cav2.1+e47 A405T, Cav2.1+e47 R1359W, Cav2.1+e47 R1667W, and Cav2.1+e47 S1799L) was then sub-cloned into pcDNA3.1(+) for functional studies. To generate the Cav2.1−e47 variants, a fragment was PCR amplified to introduce a stop codon at the exon 46–47 boundary [3]. The fragment was then substituted to the original sequence in the WT and mutant pcDNA3.1(+)-Cav2.1 plasmids to obtain Cav2.1−e47, Cav2.1−e47 A405T, Cav2.1−e47 R1359W, Cav2.1−e47 R1667W and Cav2.1−e47 S1799L. Sequence integrity was checked by sequencing the amplified fragment on both strands. For *X. laevis* oocyte injections, cDNA mixtures at 1 μg/μL with a molar ratio of 1:1:1 for Cavα:Cavβ:Cavα2δ were prepared.

### 2.2. Xenopus Oocyte Preparation and Injection

Preparation and injection of *X. laevis* oocytes were conducted as described previously [26]. The oocytes were injected with 15 nL of a solution containing the cDNA mixture of interest. Injected oocytes were maintained at 19 °C in a survival solution containing (in mM): NaCl, 96; KCl, 2; CaCl2, 1.8; MgCl2, 1; Hepes, 5; Na-pyruvate, 2.5, and gentamycin, 0.025, pH = 7.2 with NaOH, renewed daily.

### 2.3. Electrophysiology

Expressed currents were recorded at room temperature using the two-electrode voltage clamp method on day 2–3 after injection, as previously described [27]. Electrodes were pulled from borosilicate glass and filled with 3M KCl. Currents were recorded with a Geneclamp 500 amplifier (Molecular Devices, San Jose, CA, USA) and digitized with a Digidata 1200 converter (Molecular Devices) using the Clampex software (Version 7.0, Molecular Devices). The external solution (10 mM BaOH, 10 mM TEAOH, 2 mM CsOH, 50 mM N-methyl-D-glucamine, 10 mM HEPES, pH 7.2 with methane sulfonic acid) was continuously perfused in the recording chamber at the rate of 1 mL/min.

Currents were elicited by a two-pulse protocol from a holding potential of −100 mV. The first pulse lasted for 2.5 s and varied from −80 to +50 mV in 10 mV increments. The second pulse, separated from the first pulse by a 10 ms interval at −100 mV, lasted for 400 ms and was set at +10 mV. The isochronal inactivation curves were obtained by measuring the peak amplitude of the current evoked by the second pulse and by plotting the normalized current values (I/Imax) as a function of the voltage reached during the first pulse. They were fitted using the modified Boltzmann equation:

I/Imax = Rin + (1 − Rin)/(1 + exp((V − Vi)/k))

where I is the current amplitude measured during the second pulse following a first depolarization to V, Imax is the current amplitude measured during the second pulse following a first pulse at −80 mV, Vi is the half-maximal inactivation potential, k is a slope factor and Rin is the portion of non-inactivating current.

Currents were also elicited by single 400 ms-long depolarizations varying from −60 mV to +40 mV, from a holding potential of −100 mV. Current-voltage curves were obtained by measuring the peak amplitude and by plotting the normalized current values (I/Imax) as a function of the voltage. They were fitted using the modified Boltzmann equation:

I/Imax = G (V − Erev)/(1 + exp((V − Va)/k))

where I is the current amplitude measured during the depolarization to V, Imax is the peak current amplitude measured at the maximum of the current-voltage curve, G is the normalized macroscopic conductance, Erev is the apparent reversal potential, Va is the potential for half activation, and k is a slope factor. The inactivation velocity (R400) was estimated as the ratio of the peak current that disappeared after 400 ms-long depolarizations to +10 mV or 0 mV.

Recovery from inactivation was determined using a protocol with two pulses at +10 mV for 2.5 s or 100 ms, respectively, from a holding potential of −100 mV, separated by an interval of time between 100 ms and 8 s. Traces were normalized to the maximum current measured during the first pulse. The percentage of current recovered during the second pulse was plotted as a function of the inter-pulse interval. Traces were fitted with a double exponential equation.

### 2.4. Expression in HEK Cells

For immunostaining experiments, the HEK293T [28] adherent cell line (#ACC 635, DSMZ, Braunschweig Germany) was used. Cells were cultivated in high-glucose Dulbecco’s modified Eagle’s medium (DMEM; Thermo Fisher Scientific, Waltham, MA, USA) supplemented with 5% fetal bovine serum (FBS; Thermo Fisher Scientific, Waltham, MA, USA), and Penicillin-Streptamycin, in a 5% CO_2_ atmosphere at 37 °C. Cells were seeded at a concentration of 70.000 cells by cm^2^ 24 h before transient transfection. Appropriate volumes of plasmids (the total quantity of cDNA was 2 μg) and Lipofectamin^®^ reagent (Thermo Fisher Scientific, Waltham, MA, USA) were diluted in 100 µL of OptiMEM^®^ medium (Thermo Fisher Scientific, Waltham, MA, USA) and incubated for 20 min at room temperature. Complete medium exchange was carried out before transfection, and the Lipofectamin complex was added to medium. Cells were fixed with paraformaldehyde (3.7%) and kept in PBS at 4 °C for immunolabeling. Cells were treated with blocking solution (ADB, 3% BSA, 2% goat serum in PBST) for 2 h at room temperature. They were then incubated with anti-Cav2.1 (1:100, ACC-001, Alomone, Jerusalem, Israel), and anti-ZO-1 (1:100, Thermo Fisher Scientific, Waltham, MA, USA) antibodies at 4 °C overnight, washed twice with PBS 1X and then incubated with appropriate secondary antibodies for 1 h at room temperature. Cells were counterstained with 0.5 μg/mL Hoechst33342 in Vectashield^®^ antifade mounting medium (Vector Laboratories, Newark, CA, USA). Immunofluorescence was detected using an inverted Fluorescence Microscope (Montpellier Ressources Imagerie, Montpellier, France). Cav2.1 channel localization was assessed using ImageJ software. The outer and inner membrane perimeter was delineated using ZO-1 staining, and the fluorescence within the membrane and the cytoplasm were integrated as previously described [29].

### 2.5. Homology Modeling of Human Cav2.1

The model of the human channel Cav2.1 was built using the homology modeling module of BIOVIA Discovery Studio (Biovia Discovery Studio Modeling Environment, release 2021, Vélizy-Villacoublay, France). Briefly, based on the alignment of the human Cav2.1 sequence (NCBI GenBank^®^: AAB64179.1) with proteins of the Protein Data Bank [30], the structure of the human N-type voltage-gated calcium channel Cav2.2 was selected as the template (PDB ID: 7MIX-A [6]) (amino acid identity: 81.4%, and similarity: 90.4%). Twenty homology models of Cav2.1 bearing four disulfide bridges (C256-C281, C272-C287, C1406-C1417, C1771-C1782) and one calcium ion in complex were generated for the sequence: K89-D410, F469-A793, and L1233-L1939. The model with the best PDF total energy score (−43.850) was selected and evaluated with QMEANBrane to be within the expected range for a transmembrane structure [31]. Then, four mono-mutated models, A405T, R1359W, R1667W, and S1799L were generated using the Side-Chain Refinement [32] module of Discovery Studio. Details of interactions for wild and mutated residues were analyzed with the Receptor-Ligand Interactions module of BIOVIA Discovery Studio.

### 2.6. Computational Modeling

Effects of the Cav2.1 mutations on Purkinje cell firing were predicted using the Purkinje cell model developed in the NEURON simulation environment (http://www.neuron.yale.edu (accessed on 28 October 2022)) by Luthman et al. [33]. Activities of ion channels were modeled using Hodgkin-Huxley equations as previously described [33]. Six cases were analyzed here: the wild-type situation (WT Cav2.1), a hyperpolarizing shift of Va (as obtained with R1359W and R1667W mutants), a depolarizing shift of Va (as obtained with S1799L mutant), an increase of ka (as obtained with R1359W, R1667W and S1799L mutants), or both changes of Va and ka.

### 2.7. Data Analysis

Results were expressed as the mean ± SEM. The statistical significance of differences between two groups was determined using the non-paired Student’s *t*-test. Significance was set at 0.05.

## 3. Results and Discussion

### 3.1. Clinical Features Associated with A405T, R1359W, R1667W and S1799L Variants

The A405T variant (ClinVar68419) has been identified in three females from the same family with a spectrum of disorders ranging from episodic ataxia, progressive cerebellar ataxia, and familial hemiplegic migraine [34]. However, since two males carrying the same variation were healthy, it was hypothesized that additional genetic and/or environmental factors might play a role in the pathophysiology of the disease [34]. Computer-based analysis does not categorize A405T as pathogenic but rather as ‘uncertain significance’. The Cav2.1 R1359W variant (ClinVar448996) was first identified in a 20-year-old female who exhibited developmental delay, intellectual disability, epileptic seizures, and cerebellar signs [35]. The same variation was also found in a 4-year-old girl who belonged to a large cohort of patients recruited for clinical exome sequencing [36]. In both cases, the patients showed progressive cerebellar atrophy. Moreover, the R1359W variant is suspected to cause Congenital Ataxia [22], and this variation is predicted to be pathogenic or likely pathogenic by in silico algorithms. Mutation of the equivalent residue (R1358G with mouse Cav2.1 numbering) in the mouse *Cacna1a* gene has been identified in the *rolling Nagoya* mutant mice (*tg^rol^*). Homozygous *tg^rol^/tg^ro^*^l^ mice display a phenotype of severe gait ataxia and motor dysfunction [37]. Several variations of the Cav2.1 R1667 residue have been reported [38,39,40,41,42]. The R1667W (ClinVar68433), R1667Q (ClinVar585575), and R1667P (ClinVar 638582) variations are predicted to be pathogenic whereas the R1667F (ClinVar1496838) variation is of uncertain significance. The R1667W was identified during the screening for *CACNA1A* mutations in patients suffering hemiplegic migraine with or without cerebellar signs [38], in a family in which the individuals displayed progressive cerebellar ataxia either pure or associated with episodic ataxia and hemiplegic migraine [39], and in a large cohort of 412 individuals recruited to identify genetic variants in patients with hereditary cerebellar ataxia [40]. On the other hand, the R1667P variation was found in two female children with congenital ataxia and fatal cerebral edema [41] or global developmental delay [42]. The R1799L variation has been identified in an 8-year-old boy exhibiting developmental delay and showing hypotonia and ataxia [35]. The R1799L variation (ClinVar420055) and another variation of the same residue, R1799P (ClinVar624124), are predicted to be pathogenic or likely pathogenic.

### 3.2. Functional Expression of Cav2.1 Variants

The mutations were introduced into human Cav2.1 with (+e47) or without (−e47) the coding sequence of exon 47. This exon encodes the Cav2.1 cytoplasmic C-terminus and includes the CAG repeat that encodes the polyglutamine expansion associated with SCA6 [43]. The alternative splicing at the exon 46–47 boundary produces a frame-shift and introduces a premature stop codon at the beginning of exon 47, and it has been shown that Cav2.1+e47 and Cav2.1−e47 splice variants are differently affected by pathogenic mutations [44,45]. The effects of A405T, R1359W, R1667W, and S1799L substitutions on Cav2.1 channel properties were investigated after the expression of the wild type and variants with Cavβ4a and Cavα2δ1 auxiliary subunits in *X. laevis* oocytes.

**Figure 2:**
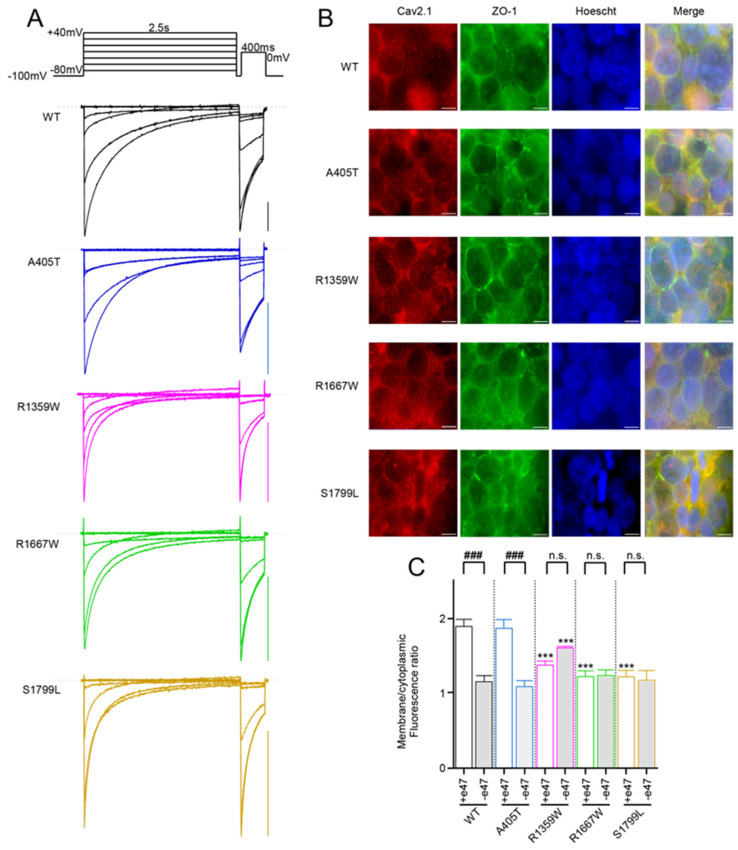
(**A**) Representative current traces were recorded from *X. laevis* oocytes that expressed the indicated Cav2.1+e47 variants with Cavβ4a and Cavα2δ1. Currents were elicited from a holding potential of −100 mV by a two-pulse protocol illustrated on top, and consisting of a 2.5 s-long depolarization from −80 mV to +40 mV, followed by a 400 ms-long depolarization to 0 mV (WT and R1359W) or +10 mV (A405T, R1667W, and S1799L). Scale bars, 200nA. (**B**) Representative images of HEK cells immunostained with anti-Cav2.1 (red) and anti-ZO-1 (green). Anti-ZO-1 was used to visualize the cell membrane. Scale bars, 10 μm. (**C**) Quantification of fluorescence intensity realized on individualized cells (*n* = 10 for each variant). Asterisks and number signs denote significant differences vs. WT (*** *p* < 0.001) and vs. Cav2.1+e47 splice variants (### *p* < 0.001), respectively (non-paired Student’s *t*-test). n.s. = non-significant.

All the Cav2.1 variants (A405T, R1359W, R1667W, and S1799L) gave rise to functional channels irrespective of the splice variant background (+e47 or −e47), and families of inward Ba^2+^ currents recorded with the Cav2.1+e47 variants are displayed on Figure 2A. Only few studies have reported a lack of expression with Cav2.1 variants carrying a missense mutation. The E1756K and F1490S mutations identified in families suffering from EA2 impair functional expression of Cav2.1 channels in heterologous expression systems [46,47]. The former mutation is located at the EEEE locus and the latter in the IIIS6 helix. More interestingly, the R174W mutation associated with Malignant Hyperthermia susceptibility ablates functional expression of Cav1.1 channels [48]. The mutation affects the innermost basic residue of the Cav1.1 IS4, similar to R1359W in the Cav2.1 IIS4. This suggests variable tolerance for the mutation between different Cavα subunits or between different S4 helices in a Cavα subunit.

Since it was virtually impossible to compare mean current amplitudes of ten different Ca^2+^ channels in the same batch of oocytes and the same day after injection due to technical constraints (cDNA injection, inhibition of endogenous Cl^−^ current with BAPTA), we evaluated the effects of the mutations on Cav2.1 membrane expression by immunostaining of HEK cells transfected with WT or mutant channels together with Cavβ4a and Cavα2δ1 subunits (Figure 2B). We measured a weaker membranous expression for Cav2.1−e47 compared to Cav2.1+e47 (Figure 2C), reminiscent of previous observations realized in HEK cells [45]. This decrease was also found for Cav2.1 splice variants containing the A405T substitution but not the R1359W, R1667W, or S1799L substitutions (Figure 2C). Compared to WT channels, R1667W and S1799L induced a weaker membranous expression with the Cav2.1+e47 splice variant but not with the Cav2.1−e47 splice variant, and R1359W induced a weaker or a stronger expression in the Cav2.1+e47 or Cav2.1−e47 slice variant background, respectively. In most cases, Cav2.1 pathogenic mutations decrease current density (but see A713T in IIS6 [29] for counterexample), and the reduced influx of Ca^2+^ during membrane depolarization may be involved in the pathophysiological mechanism of the disease. The decrease of current density can be linked to a weaker expression of channels at the plasma membrane (for example G230 in IS5 helix [29]), or to a biophysical effect altering channel conductance (for example Y1384C in IIIS6 helix [45]). However, it is noteworthy that current density can vary between heterologous expression systems and neuronal cells, and even between different types of neurons (R592Q in IS4 [49,50]). Moreover, we show here that Cav2.1 membranous expression, at least in HEK cells, is splice variant-dependent (Figure 2C). It is important to keep these observations in mind to avoid over-interpretation. That said, our results show that the studied mutations, as many others, influence Cav2.1 channel expression at the cell membrane.

### 3.3. Functional Characterization of Cav2.1+e47 and Cav2.1−e47

**Figure 3:**
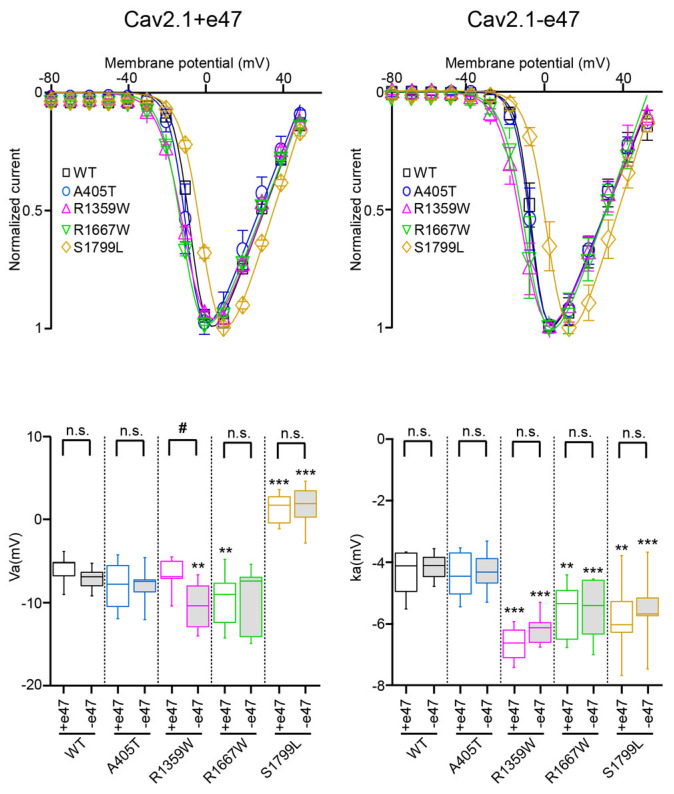
Voltage dependence of Cav2.1 activation. (**Top**) Average current-voltage relationships for *X. laevis* oocytes expressing Cav2.1+47 (**left**) or Cav2.1−e47 variants (**right**). (**Bottom**) Box plots of half-maximal activation potential (Va, left) and slope factor of the Boltzmann curve of channel activation (ka, right) were obtained for all Cav2.1 variants studied. The mean values are given in Table 1. Asterisks and number signs denote significant differences vs. WT (** *p* < 0.01, *** *p* < 0.001) and vs. Cav2.1+e47 splice variant (# *p* < 0.05), respectively (non-paired Student’s *t*-test). n.s. = non-significant.

**Figure 4:**
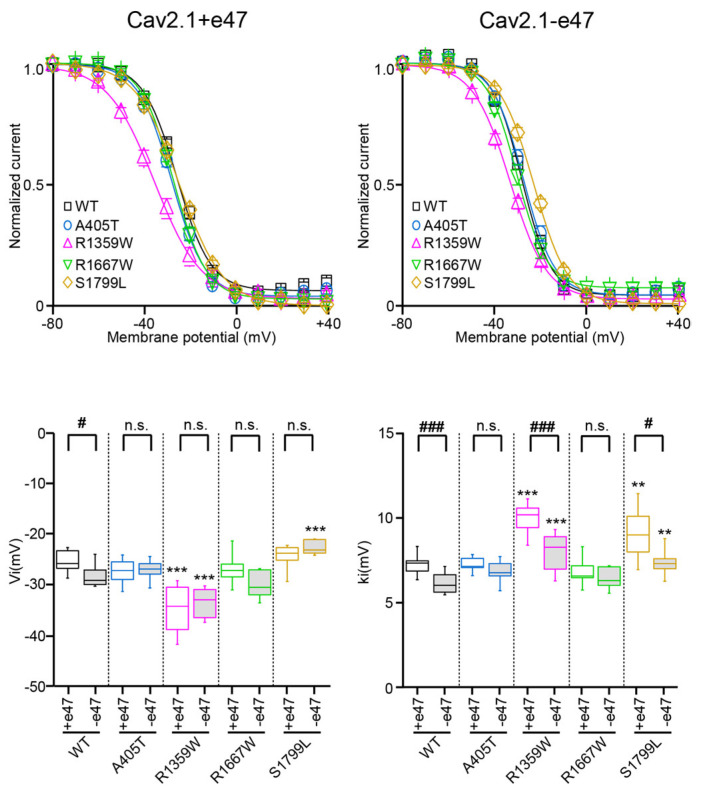
Voltage dependence of Cav2.1 inactivation. (**Top**) Voltage-dependent isochronal inactivation curves for *X. laevis* oocytes expressing Cav2.1+47 (**left**) or Cav2.1−e47 (**right**) variants. (**Bottom**) Box plots of half-maximal inactivation potential (Vi, left) and slope factor of the Boltzmann curve of channel inactivation (ki, right) were obtained for all Cav2.1 variants studied. The mean values are given in Table 1. Asterisks and number signs denote significant differences vs. WT (** *p* < 0.01, *** *p* < 0.001) and vs. Cav2.1+e47 (# *p* < 0.05, ### *p* < 0.001), respectively (non-paired Student’s *t*-test). n.s. = non-significant.

**Figure 5:**
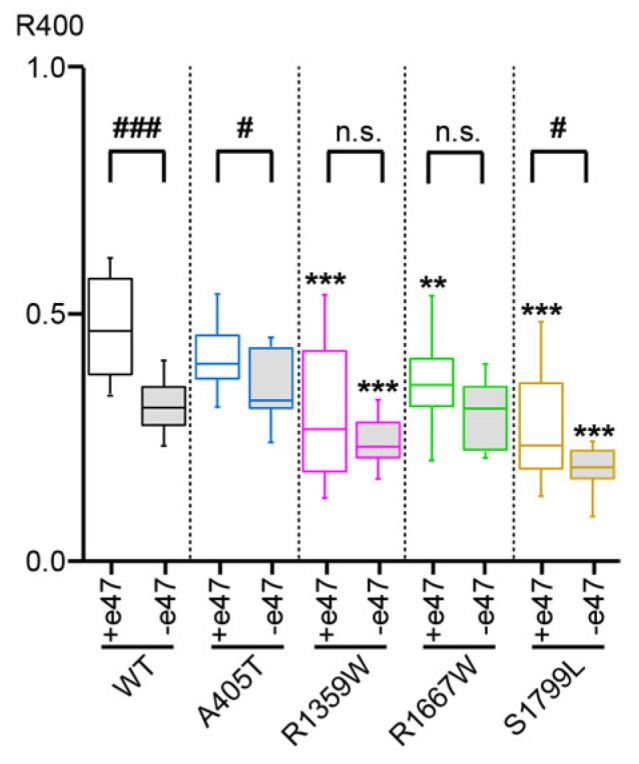
Cav2.1 inactivation kinetics. Box plot of the ratio of remaining current at the end of a 400 ms-long depolarization (R400) to 0 mV (WT, A405T, R1359W, and R1667W Cav2.1 variants) or + 10 mV (S1799L Cav2.1 variants) with respect to the peak current amplitude. Asterisks and number signs denote significant differences vs. WT (** *p* < 0.01, *** *p* < 0.001), and vs. Cav2.1+e47 (# *p* < 0.05, ### *p* < 0.001), respectively (non-paired Student’s *t*-test). n.s. = non-significant.

**Figure 6:**
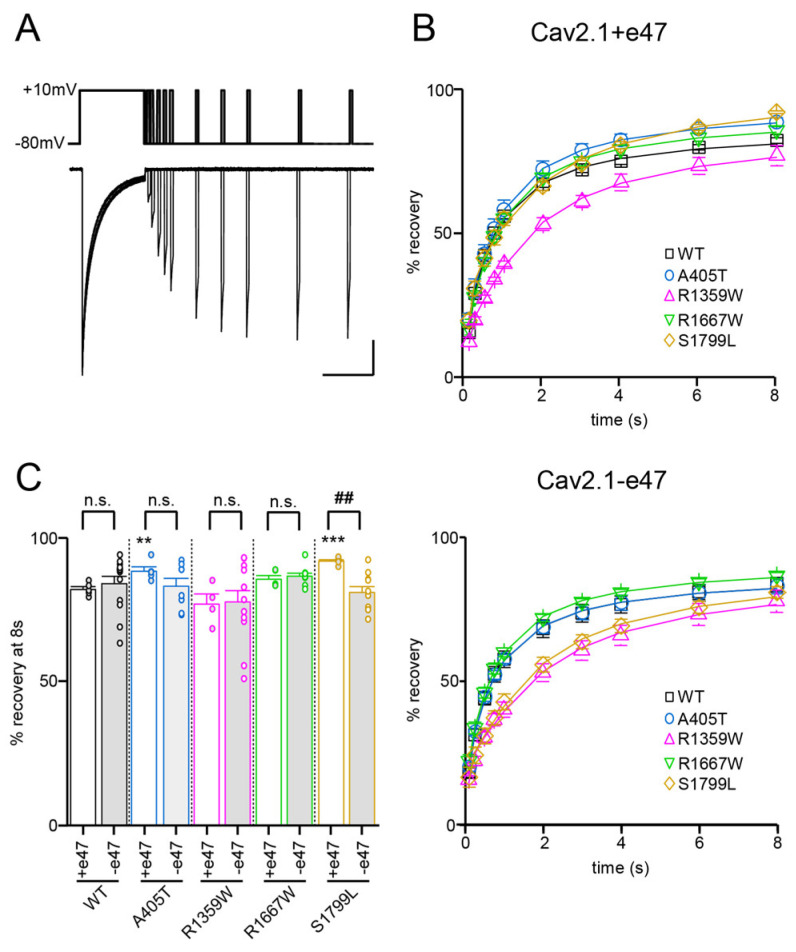
Recovery from inactivation of Cav2.1 variants. (**A**) Representative current traces were obtained in *X. laevis* oocytes expressing Cav2.1+47 with Cavβ4a and Cavα2δ1. The experimental protocol, illustrated above traces, consists of a 2.5 s-long depolarization to +10 mV, followed by inter-pulse intervals between 100 ms and 8 s, and a second 100 ms-long depolarization to +10 mV. Scale bars: 200 nA and 2 s. (**B**) Percentage of current recovery plotted against the inter-pulse duration for Cav2.1+e47 (**top**) and Cav2.1−e47 variants (**bottom**). (**C**) Bar graph showing percent recovery at 8 s for all Cav2.1 variants studied. Asterisks and number signs denote significant differences vs. WT (** *p* < 0.01, *** *p* < 0.01) and vs. Cav2.1+e47 (## *p* < 0.01), respectively (non-paired Student’s *t*-test). n.s. = non-significant.

We then compared the voltage-dependent properties of the different channels starting with wild-type channels. Both Cav2.1+e47 and Cav2.1−e47 possessed similar half-maximal activation potentials (Va = −6.0 ± 0.6 mV, *n* = 12, and −7.2 ± 1.6 mV, *n* = 12, respectively, Figure 3 and Table 1), and similar slope factors of the Boltzmann curves of channel activation (ka = −4.5 ± 0.2 mV, *n* = 12, and −4.2 ± 0.5 mV, *n* = 12, respectively). However, the presence of exon 47 induced a small depolarizing shift of the half-maximal inactivation potential, and significantly increased the slope factor of the Boltzmann curve of channel inactivation (Vi = −25.8 ± 0.6 mV and −28.2 ± 0.7, *p* < 0.05, and ki = 7.3 ± 0.2 mV and 6.2 ± 0.2 mV, *p* < 0.001, for Cav2.1+e47, *n* = 10, and Cav2.1−e47, *n* = 13, respectively, Figure 4 and Table 1). These results are in agreement with those we obtained in similar recording conditions with the rat Cav2.1+e47 and Cav2.1−e47 variants [51]. However, they differ from those obtained with human variants expressed in HEK cells, since in previous studies, both Cav2.1+e47 and Cav2.1−e47 displayed similar voltage-dependence of activation and inactivation [44,45]. We estimated the inactivation rate during a pulse by measuring the ratio of current remaining after a 400 ms-long depolarization (R400, Figure 5), and found that Cav2.1+e47 currents decayed significantly more slowly compared to Cav2.1−e47 (R400 = 0.47 ± 0.02 *n* = 22, and 0.32 ± 0.02, *n* = 17, respectively; *p* < 0.001, Table 2). Finally, we also compared the recovery from inactivation between pulses by using a two-pulse protocol separated by a time interval ranging from 100 ms to 8 s (Figure 6). Rates of recovery from inactivation were well fitted with double exponential equation, and the time constants obtained for Cav2.1+e47 and Cav2.1−e47 were similar (τau1 = 115 ± 10 ms and τau2 = 1062 ± 31 ms, *n* = 5, and τau1 = 119 ± 22 ms and τau2 = 1092 ± 182 ms, *n* = 11, respectively). Both channels displayed also a similar recovery at 8 s (82 ± 1%, *n*= 5, and 85 ± 2%, *n* = 11; Figure 6 and Table 2). Although Cav2.1−e47 inactivated faster than Cav2.1+47, both channels recovered from inactivation at the same rate. Again, our results differ from previous studies in which inactivation kinetics of Cav2.1+e47 and Cav2.1−e47 were similar, and recovery from inactivation of Cav2.1+e47 was different from that of Cav2.1−e47 [44,45]. Unlike different heterologous expression systems (*X. laevis* oocytes instead of HEK cells), the Cav2.1 clone that we used lacked the ‘VEA’ insertion in the II-III linker, the ‘NP’ insertion in the IVS4-S5 linker, and more interestingly possessed the alternative exon 37a compared to the Cav2.1 clone used in the above-mentioned studies. Exon 36 and one of the two mutually exclusive exons 37a or 37b encode the EF hand-like domain in the Cav2.1 carboxy terminal: either EFa or EFb [3]. EF-hand influences Cav2.1 gating properties in an exon 47-dependent manner [52]. It is therefore likely that the different sequence at exon 37 may be the cause of the slight differences between our results and those of Adams et al. [44] and Gandini et al. [45], especially concerning the different rates of recovery from inactivation. This possibility will be the subject of future investigations.

### 3.4. Functional Characterization of Cav2.1 A405T Variants

Compared to wild-type channels, the A405T substitution did not produce any significant effect on the voltage dependence of activation (Cav2.1+e47 A405: Va = −7.7 ± 0.8 mV and ka = −4.4 ± 0.2 mV, *n* = 14; Cav2.1−e47 A405T: Va = −7.9 ± 2.3 mV and ka = −4.3 ± 0.7 mV, *n* = 8; Figure 3 and Table 1) and inactivation (Cav2.1+e47 A405T: Vi = −27.8 ± 0.9 mV and ki = 7.1 ± 0.2 mV; Cav2.1−e47 A405T: Vi = −27.5 ± 0.9 mV and ki = 6.7 ± 0.3 mV, *n* = 7; Figure 4 and Table 1). Mutants displayed similar inactivation kinetics than WT channels (R400 = 0.41 ± 0.01, *n* = 28 and 0.35 ± 0.02, *n* = 11 for Cav2.1+e47 A405T and Cav2.1−e47 A405T, respectively; Figure 5 and Table 2), and a faster recovery from inactivation was found only in the Cav2.1+e47 splice variant background (Cav2.1+e47 A405T: 88 ± 2%, *n* = 5, *p* < 0.01 vs. WT; Cav2.1−e47: 83 ± 3%, *n* = 8; Figure 6 and Table 2). The A405 residue is located in the Cavα I-II linker close to the Alpha Interaction Domain (AID), which is involved in the interaction between the Cavα and the Cavβ subunits [53]. The Cavβ subunit allows membrane trafficking of Cavα [54] and is an important regulator of the Cavα properties, the different Cavβ subunits producing specific effects [26]. Since the A405T substitution does not affect Cav2.1 expression and voltage-dependent properties, we can conclude that this mutation does not modify the Cavα/β interaction, nor the structural rearrangements induced by Cavβ which modulate Cavα properties.

Given the presence of healthy carriers in the family in which the A405T mutation was found [34], the lack of noticeable effects on Cav2.1 properties is not unexpected. However, it questions the pathophysiological mechanism of the disorders found in the three individuals of the family. Interestingly, another and similar mutation in the Cav2.1 I-II loop, A454T (ClinVar68420, categorized as pathogenic, or likely pathogenic), has been found in two sisters with progressive ataxia [55], and more intriguingly, in a family of migraineurs (including FHM) in which it appeared that A454T substitution was not the cause of migraine but modified the disease phenotype [56]. In HEK cells, voltage-dependent activation (Va, ka), inactivation kinetics, and recovery from inactivation of Cav2.1 A454T variant were similar to the WT channel; however, Vi was shifted towards hyperpolarized or depolarized potentials depending on which Cavβ (Cavβ2 or Cavβ3) subunit was co-expressed with the mutant channel [56]. Moreover, A454T mutation decreased Cav2.1 modulation by Syntaxin1A or SNAP25, two members of the protein family mediating vesicle docking and fusion (SNAREs) which are known to interact with the intracellular linker connecting repeat II and III [57]. Even if phenotypes associated with A454T mutation differ from those associated with A405T mutation, a similar modification of the regulation by different Cavβ subunits (Cavβ1 and Cavβ2 instead of Cavβ4) or by SNARE could not be excluded, but these possibilities were not assessed in the present study.

### 3.5. Functional and Structural Analysis of R1359W and R1667W Variants

The R1359W and R1667W substitutions involve similar residues but are located in the repeat III or IV, respectively, and produced distinct effects when introduced in Cav2.1+e47 or Cav2.1−e47. Both mutations induced a hyperpolarizing shift of Va in a splice variant-dependent manner (Cav2.1+e47 R1359W: −7.9 ± 0.9 mV, *n* = 12; Cav2.1−e47 R1359W: −10.7 ± 2.8 mV, *n* = 10, *p* < 0.01 vs. WT; Cav2.1+e47 R1667: −9.5 ± 0.7 mV, *n* = 21, *p* < 0.01 vs. WT; Cav2.1−e47 R1667W: −9.7 ± 3.9 mV, *n* = 6), and increased ka whatever the splice variant background (Cav2.1+e47 R1359W: −6.6 ± 0.2 mV, *n* = 12, *p* < 0.001 vs. WT; Cav2.1−e47 R1359W: −6.2 ± 0.5 mV, *n* = 11, *p* < 0.001 vs. WT; Cav2.1+e47 R1667W: −5.6 ± 0.2, *n* = 21, *p* < 0.01 vs. WT; Cav2.1−e47 R1667W: −5.6 ± 1.0 mV, *n* = 6, *p* < 0.001 vs. WT; Figure 3 and Table 1). The R1359W and R1667W also behaved differently regarding the voltage dependence of inactivation. Indeed, in both Cav2.1 splice variants, R1667 substitution did not affect either Vi or ki (Cav2.1+e47 R1667: Vi = −26.9 ± 0.8 mV and ki = 6.8 ± 0.2 mV, *n* = 15; Cav2.1−e47 R1667W: Vi = −30.0 ± 1.1 mV and ki = 6.4 ± 0.2 mV; *n* = 7). In strike contrast, in both Cav2.1 splice variants, R1359W substitution induced a hyperpolarizing shift of Vi (Cav2.1+e47 R1359W: −34.8 ± 1.4, *n* = 13, *p* < 0.001 vs. WT; Cav2.1−e47 R1359W: −33.6 ± 0.9 mV, *n* = 12, *p* < 0.001 vs. WT) and an increase of ki (Cav2.1+e47 R1359W: 9.9 ± 0.3, *n* = 13, *p* < 0.001 vs. WT; Cav2.1−e47 R1359W: 8.0 ± 0.4 mV, *n* = 12, *p* < 0.001 vs. WT; Figure 4 and Table 1). The R1359W and R1667W mutations accelerated inactivation kinetics in both Cav2.1 splice variants in the case of R1359W (Cav2.1+e47 R1359W: 0.31 ± 0.03, *n* = 29, *p* < 0.001 vs. WT; Cav2.1−e47 R1359W: 0.24 ± 0.01, *n* = 18, *p* < 0.001), and in a splice variant-dependent manner in the case of R1667W (Cav2.1+e47 R1667W: 0.36 ± 0.02, *n* = 44, *p* < 0.01 vs. WT; Cav2.1−e47 R1667W: 0.30 ± 0.02, *n* = 13; Figure 5 and Table 2). Finally, R1667W recovered from inactivation similarly to WT channels (Cav2.1+e47 R1667W: 85 ± 1%, *n* = 5; Cav2.1−e47 R1667W: 86 ± 1%, *n* = 9), whereas R1359W recovered more slowly. This slow recovery is obvious in Figure 6 for both Cav2.1 splice variants (Cav2.1−e47 R1359W: τau1 = 100 ± 9 ms and τau2 = 1697 ± 80 ms, *n*= 4, *p* < 0.001 vs. WT; Cav2.1+e47 R1359W: τau1 = 129 ± 32 and τau2 = 2125 ± 270 ms, *n* = 12, *p* < 0.01 vs. WT). The long recovery from inactivation can lead to profound current attenuation during bursts of depolarization. Nevertheless, the recovery at 8 s of both Cav2.1+e47 R1359W and Cav2.1−e47 R1359W was similar to that of WT channels (77 ± 4%, *n* = 4 and 78 ± 4%, *n* = 12, respectively; Figure 6 and Table 2). However, we can expect that Cav2.1 R1359W accumulates in the inactivated state during rapid firing conditions.

Overall, the hyperpolarizing shift of Vi (Figure 4), the faster inactivation kinetics (Figure 5), and the decreased rate of recovery from inactivation (Figure 6) observed with R1359W seem to indicate a stabilization of the inactivated state. R1359W may be therefore defined as a loss-of-function mutation. On the other hand, R1667W causes both channel gain-of-function (hyperpolarizing shift of Va, Figure 3) and loss-of-function (faster inactivation kinetics, Figure 5) in a splice variant-dependent manner. Therefore, our results show that R1667W, like many other pathological mutations (see [42] and references included), cannot be strictly defined as a channel gain-of-function or loss-of-function.

**Figure 7:**
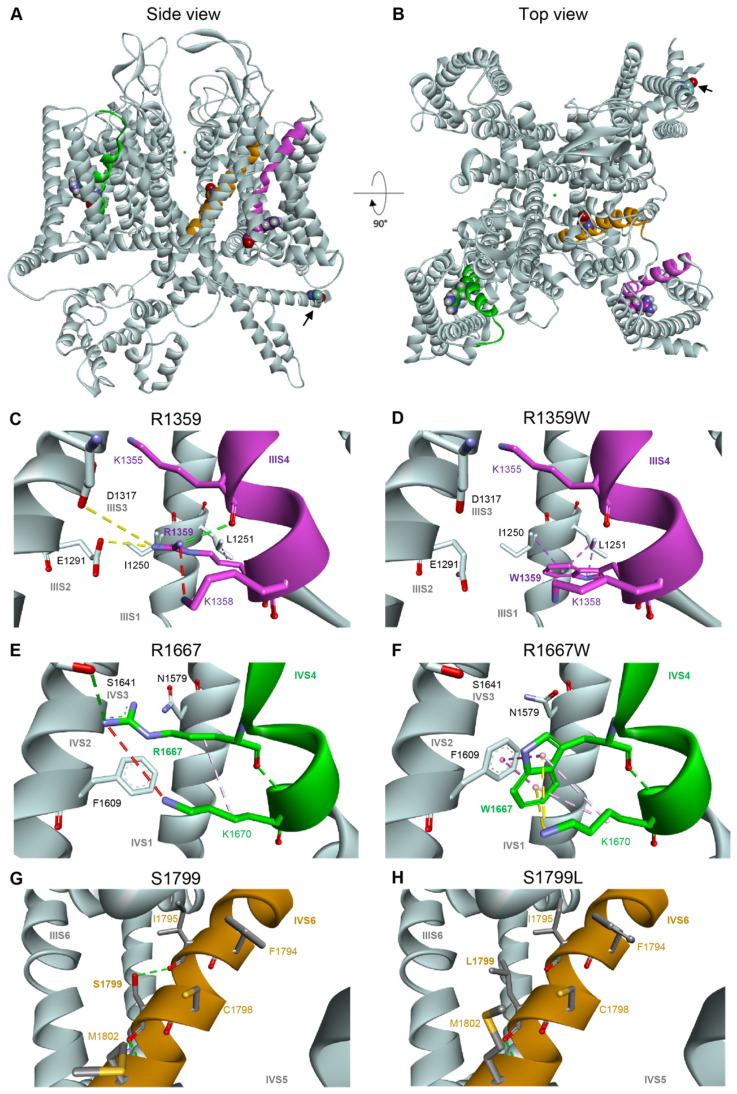
Homology model of human Cav2.1 viewed from the side (**A**) or from the top (**B**). The IIIS4, IVS4, and IVS6 transmembrane helices carrying the studied mutation are represented in magenta, dark green, and brown, respectively. The four residues R1359 (IIIS4), R1667 (IVS4), S1799L (IVS6), and A405 (I-II loop, indicated with black arrow) are represented in balls. (**C**,**D**) Modeling of the voltage sensing domain (VSD) in repeat III of WT Cav2.1 (**C**), and Cav2.1 R1359W (**D**), showing putative non-bonded interactions in dash lines: hydrogen bond pairs in green (K1355 in IIIS4), ionic bond pairs in yellow (E1291 in IIIS2, and to a lesser extent, D1317 in IIIS3), hydrophobic interaction in purple (L1251 in IIIS1). Steric clashes (K1358 in IIIS4) are represented by a red dashed line. (**E**,**F**) Modeling of the IV-VSD of WT Cav2.1 (**E**), and Cav2.1 R1667W (**F**). (**G**,**H**) Modeling of the pore domain around the IVS6 helix of WT Cav2.1 (**G**), and Cav2.1 S1799L (**H**).

To analyze the functional impact of the mutations on Cav2.1 channel structure, we generate a homology model based on the atomic resolution structure of human Cav2.2 displaying a closed pore, and the IS4, IIIS4, and IVS4 helices in the depolarized state (S4s are ‘up’), and the IIS4 helix exhibiting a ‘down’ conformation [6]. R1359 and R1667 are perfectly conserved in all voltage-gated Ca^2+^ channels [22], and R1359 is the IIIS4 innermost gating charge (R5) while R1667 is in the midst of IVS4 (R3) (Figure 7A). R1359 residue is attracted towards S2 and S3 helices thanks to electrostatic interactions with E1291 (IIIS2) and D1317 (IIIS3), and a repulsive interaction with the neighboring K1358 (IIIS4) (Figure 7C). The salt bridges are lost upon replacement of the arginine residue with the neutral tryptophane (Figure 7D). Indeed, the novel W1359 residue lies between hydrophobic interactions with I1250 and L1251 (IIIS1), and a potential pi-cation interaction with K1358. These new interactions impact probably the mobility of the IIIS4, which may explain the stabilization of the inactivated state. As noted previously [42], R1667 is positioned just extracellular to F1609 (IVS2), which constitutes the VSDII hydrophobic constriction site that prevents transmembrane movement of water and ions [58]. R1667 makes hydrogen bonds with S1641 (IVS3) and N1579 (IVS1), potentially reinforced by repulsive interaction with K1670 (IVS4) (Figure 7E). The interactions are lost upon R1667 substitution, and the novel W1667 residue is surrounded by highly stabilizing hydrophobic (K1670) and pi-cation (F1609) interactions (Figure 7F).

Pathogenic mutations affecting the gating charges can be found in the four Cav2.1 S4 helices (see Figure 1 for IIIS4 and IVS4 helices). One of the first mutations associated with FHM1 was an R192Q substitution of the outermost (R1) basic residue of the IS4 helix [59]. The functional consequences of this specific Cav2.1 mutation have been the subject of several studies, using heterologous expression system [44,60,61,62], transfected hippocampal [63] or trigeminal ganglion [64] neurons from Cav2.1 knockout mice, and moreover, an R192Q knockin mouse strain has been generated [50]. In *X. laevis* oocytes, the R192Q mutation did not change the Cav2.1 voltage dependence of activation and inactivation [60], and the Cav2.1 voltage dependence of activation is affected by R192Q mutation only in a subpopulation of trigeminal ganglion neurons [50]. However, in HEK cells, the R192Q substitution in Cav2.1 produced a hyperpolarizing shift of both Va and Vi and did not modify ka and ki [44]. Similar effects were produced by other substitutions: R582Q (R2 in IIS4), associated with FHM1 [65], and R1349Q (R2 in IIIS4, Figure 1) found in the *tg-5J* ataxic mouse strain [66]. In our study, R1359W (IIIS4) displaced also Va and Vi towards hyperpolarized voltages, but in a splice variant-dependent manner, and increased both ka and ki (Figure 3 and Figure 4). In strike contrast, mutations of basic residues R3 (R1667P [42] and R1667W (this study)) or R5 (R1679P [67]) in the IVS4 helix, did not modify voltage dependence of inactivation (Vi and ki), although they produced an increased in ka and either a hyperpolarizing (R3 mutations) or a depolarizing (R5 mutation) shift of Va. Some discrepancies found between studies arise undoubtedly from the different Cav2.1 variants and/or the different heterologous expression systems, but they also suggest that equivalent substitutions in different S4 helices or different mutations of the same residue induce specific alterations of the channel gating properties. Functional differences between the four S4 helices of the Cavα subunit are suggested by the specific length of the helix and/or the various number of charged residues in each S4 (see Figure 1 for IIIS4 and IVS4) and are demonstrated by accumulating evidence obtained with Cav1 channels [68]. Neutralization of S4 charges revealed the critical role played by IS4 and IIIS4 helices in Cav1.2 channel activation whereas IIS4 and IVS4 helices have less or no impact [69]. Charged residues in helix IS4 are also critical for the voltage dependence of Cav1.2 inactivation, whereas neutralization of charges in IIS4 and IIIS4 has less effect [70] (see also [4]). Using voltage-clamp fluorometry, which allows the tracking of individual S4 movement, Pantazis et al. [71] found that the four Cav1.2 S4 helices exhibit distinct voltage-dependent properties and that IIS4 and IIIS4 helices are critical for channel activation. Using the same approach, Savalli et al. [72] highlighted the prominent role played by IS4 in Cav1.1 activation. Similar studies on Cav2 channels are lacking, and it is highly speculative to think that the biophysical mechanisms leading to channel activation are strictly the same for Cav1 and Cav2 channels. The charged residues in S4 helices of Cav1 and Cav2 channels are not perfectly conserved, and the cryo-electron microscopy of Cav1.1 [73,74] and Cav2.2 [6,7] has highlighted fundamental differences between the VSDs of the two channels. Nevertheless, studies with pathogenic variants (including ours) suggest that S4 helices of the four repeats are involved in the voltage dependence of Cav2.1 activation, whereas only the IS4, IIS4, and IIIS4 helices are involved in the voltage dependence of channel inactivation.

### 3.6. Functional and Structural Analysis of S1799L Variants

The last mutation studied herein, S1799L, is located in the midst of the IVS6 helix. The S1799L substitution induced a depolarizing shift of Va and increased ka, whatever the splice variant background (Cav2.1+e47 S1799L: Va = 1.2 ± 0.5 mV, *p* < 0.001 vs. WT, and ka = −5.8 ± 0.3 mV, *p* < 0.01 vs. WT, *n* = 15; Cav2.1−e47 S1799L: Va = 1.7 ± 2.5 mV, *p* < 0.001 vs. WT, and ka = −5.5 ± 0.1 mV, *p* < 0.001 vs. WT, *n* = 9; Figure 3 and Table 1). The mutation produced a depolarizing shift of Vi only in Cav2.1−e47 (Cav2.1+e47 S1799L: −24.5 ± 0.7 mV, *n* = 13; Cav2.1−e47 S1799L: −22.7 ± 0.6 mV, *n* = 6, *p* < 0.001 vs. WT), and increased ki in both Cav2.1+e47 and Cav2.1−e47 splice variants (9.1 ± 0.5 mV, *n* = 13, *p* < 0.01 vs. WT, and 7.4 ± 0.3 mV, *n* = 6, *p* < 0.01 vs. WT, respectively; Figure 4 and Table 1). The inactivation kinetics were faster (Cav2.1+e47 S1799L: 0.27 ± 0.02, *n* = 38, *p* < 0.001; Cav2.1−e47 S1799L: 0.19 ± 0.02, *n* = 13, *p* < 0.001; Figure 5 and Table 2), and the recovery from inactivation were slower (Cav2.1+e47 S1799L: τau1 = 105 ± 14 ms and τau2 = 1886 ± 133 ms, *n* = 6, *p* < 0.001 vs. WT; Cav2.1−e47 S1799L: τau1 = 158 ± 42 ms and τau2 = 2092 ± 247 ms, *n* = 10, *p* < 0.01 vs. WT) in any Cav2.1 splice variants. Nevertheless, the recovery at 8 s is either higher than or similar to that of WT depending on the splice variant background (Cav2.1+e47 S1799L: 92 ± 1%, *n* = 6, *p* < 0.001 vs. WT; Cav2.1−e47 S1799L: 81 ± 2%, *n* = 10; Figure 6 and Table 2). Understanding the functional effect produced by S1799L mutation in IVS6 is challenging since, like R1667W, it causes channel loss-of-function (depolarizing shift of Va, and faster inactivation kinetics) and gain-of-function but in a splice variant-dependent manner (depolarizing shift of Vi and recovery from inactivation). Homology modeling reveals that S1799 makes a single hydrogen bond with I1795, which is lost upon S1799L substitution. This lack of obvious structural changes induced by a pathogenic mutation in the Cav2.1 pore domain has been previously observed [29]. The wide opening of the activation gate may result from the subtle movements of the S6 segments [8]. Nevertheless, these rearrangements likely involve multiple interactions that stabilize the various states of the channel while opening/closing, and S6 pathogenic mutations, such as S1799L, may impact states that are not predicted by homology modeling.

In striking contrast to VSDs, the intracellular gate formed by S6 helices is nearly identical in Cav1.1 and Cav2.2 structures [6,7,73,74], and insights on Cav2 structure-function relationships might come from pathological mutations found on Cav1 channels. The Cav1.4 I754T variant is associated with a severe visual disorder and induces a remarkable hyperpolarizing shift of Va and a significant decrease in inactivation kinetics [75]. The residue, located at the cytoplasmic end of IIS6, is perfectly conserved in all Cavα subunits (I711 in Cav2.1), and, when introduced to Cav1.2, the substitution produces the same effects as in Cav1.4 [76]. On the other hand, Timothy syndrome is a multi-systemic disorder with dysmorphic features, long-QT syndrome, and autism spectrum disorders caused by a mutation in Cav1.2 channels [77]. The first mutation associated with Timothy syndrome, G406R in IS6, produces a hyperpolarizing shift of half-maximal activation potential and a dramatic decrease in inactivation kinetics. Again, this residue is perfectly conserved in all Cavα subunits (G361 in Cav2.1), and when introduced in Cav2.3 [78] or Cav2.1 [27], the effects produced by the mutation are similar to those produced in Cav1.2. These two examples suggest that the pore of both Cav1 and Cav2 share common steps during channel activation and inactivation. Until now, it has been difficult to have functional insights into channel gating from studies focused on Cav2.1 pathogenic mutations located in S5 and S6 helices. Indeed, several Cav2.1 variants, associated with FMH1 (V714A [29,60,63,79] and D715E in IIS6 [65], V1695I in IVS5 [80], and I1810L in IVS6 [60,61,81]), with Epileptic Encephalopathy (G230V in IS5, A713T in IIS6 and V1396M in IIIS5 [29]), with episodic ataxia and epilepsy (L621R in IIS5 [82]), and with hemiplegic migraine and developmental delay (Y1384C in IIIS5 [45]) are located in the pore domain and have been studied in heterologous expression systems or transfected neurons. Given the diversity of phenotypes, residue location in helix, and their substitution, the functional consequences of these mutations on the voltage dependence of activation and inactivation are very different. Moreover, the decrease of Ca^2+^ influx caused by the weaker expression of functional channels at the plasma membrane and/or by the reduced conductance of the mutated pore might cause the pathophysiology without any modification of voltage-dependent properties. Nevertheless, these disease-causing mutations in the pore domain uncover the structural rearrangements undergone by S5 and S6 helices upon channel activation and inactivation.

### 3.7. Computational Simulation

**Figure 8:**
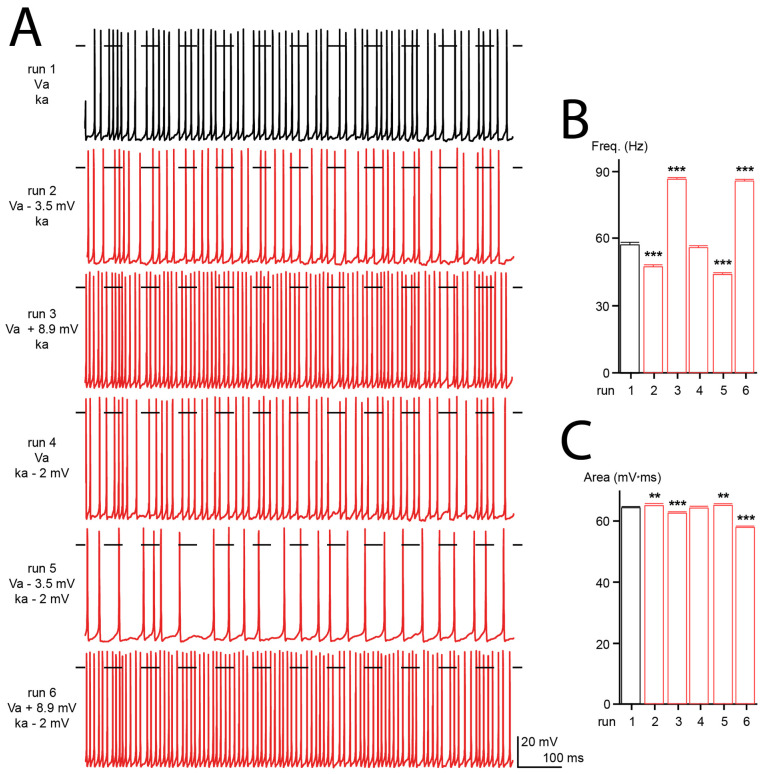
Computer modeling of human Purkinje cell. (**A**) Representative firing patterns obtained from simulated Purkinje cell with WT Cav2.1 properties (Va, ka, run 1), with a hyperpolarizing shift of Va (run 2), a depolarization shift of Va (run 3), an increase of ka (run 4), or a combination of both (run 5 and 6). (**B**) Bar graph showing the mean action potential frequencies (freq) obtained for the different simulations (in Hertz: 57.3 ± 0.8, 47.3 ± 1.0, 86.3 ± 0.7, 55.7 ± 0.9, 43.8 ± 0.8 and 85.5 ± 0.7 for run 1 to run 6, respectively). (**C**) Bar graph showing the mean action potential areas obtained for the different simulations (in mV.ms: 64.0 ± 0.1, 64.7 ± 0.2, 62.2 ± 0.1, 64.0 ± 0.1, 64.8 ± 0.2, 57.6 ± 0.1 for run 1 to 6, respectively). The number of action potentials analyzed was between 389 and 807. Asterisks denote significant difference vs. run 1 (** *p* < 0.01, *** *p* < 0.01) (non-paired Student’s *t*-test).

Cav2.1 channels are the predominant Ca^2+^ channels expressed in Purkinje cells, where they play a fundamental role in cellular excitability [83,84]. Purkinje cells fire spontaneously, and their activity is required to perform planned and coordinated movements. Consequently, malfunction of Purkinje cells often results in motor dysfunctions [85]. To seek the effects of Cav2.1 mutations on neuronal electrical activity, we used a computer model of the human Purkinje cell where all the ionic conductances were modeled with the Hodgkin and Huxley formalism [33]. Six simulations were run with: the wild-type electrophysiological conductances (run 1), a 3.5 mV hyperpolarizing shift of Va (as found with the R1359W variant, run 2), an 8.9 mV depolarizing shift of Va (as found with the S1799L variant, run 3), a 2 mV increase of ka (as found with the R1359W variant, run 4), and both a hyperpolarizing shift of Va and increase of ka (run 5) or a depolarizing shift of Va and increase of ka (run 6) (Figure 8A). We did not observe any modification of action potential frequency and area upon decreasing the steepness of ka (Figure 8B,C). However, the hyperpolarizing shift of Va decreased the frequency and increased the area, while the depolarizing shift produced the opposite effect (Figure 8B,C). Area changes resulted from a modification of the rates of the rising or decaying phases of action potential (not shown). A modification of frequency may be explained by the variation of the Ca^2+^ influx caused by the Va shift, and consequently an altered amplitude of the KCa was induced after hyperpolarization [85]. The additional modification of ka tends to increase the effects produced by Va changes (Figure 8B,C). These results suggest that the biophysical changes produced by Cav2.1 pathogenic mutations, such as those studied herein, can dramatically alter the firing pattern of neuronal cells.

## 4. Conclusions

Next-generation sequencing has allowed the identification of hundreds of variations in *CACNA1A*. Their impact on Cav2.1 biophysical properties has been studied for only a few of them, occasionally highlighting discrepancies in the phenotype/genotype correlation. Nevertheless, such studies are necessary to elucidate the pathophysiological mechanism of the diseases associated with Cav2.1 mutations. Moreover, they shed new light on structure/function relationships and may help to understand the molecular rearrangements that lead to channel activation and inactivation. Of the four Cav2.1 mutations studied herein, which were associated with distinct phenotypes, one did not modify the biophysical properties of the channel (A405T), one stabilizes the inactivated state (R1359W), and the last two produce both the channel lost-of-function and gain-of-function in a splice variant-dependent manner (R1667W and S1799L). Simulations suggest that altered biophysical properties may affect the firing of Purkinje cells. Apart from the Cav2.1 Ca^2+^ channel, K^+^ channels also play a crucial role in pacemaking activity [15,86], and it has been shown that the K^+^ channel blocker 4-AP was able to restore the precision of Purkinje cell pacemaking in the *tottering* mice [87] and in a mouse model of SCA6 [88]. Moreover, the Ca^2+^-dependent K^+^ channel activators EBIO and chlorzoxazone can rescue the firing regularity of Purkinje cells in *leaner*, *ducky,* and *tottering* mice [83,87]. We may therefore expect that future therapies targeting Cav2.1 channels would be as effective for improving motor functions in neurodevelopmental diseases [89].

## Figures and Tables

**Table 1:** Voltage dependence of Cav2.1 activation and inactivation.

		Va (mV)	ka (mV)	*n*	Vi (mV)	ki (mV)	*n*
WT	+e47	−6.0 ± 0.6	−4.5 ± 0.2	12	−25.8 ± 0.6	7.3 ± 0.2	9
	−e47	−7.2 ± 1.6	−4.2 ± 0.5	12	−28.2 ± 0.7 #	6.2 ± 0.2 ###	13
A405T	+e47	−7.7 ± 0.8	−4.4 ± 0.2	14	−27.8 ± 0.9	7.1 ± 0.2	11
	−e47	−7.9 ± 2.3	−4.3 ± 0.7	8	−27.5 ± 0.9	6.7 ± 0.3	7
R1359W	+e47	−7.9 ± 0.9	−6.6 ± 0.2 ***	12	−34.8 ± 1.4 ***	9.9 ± 0.3 ***	12
	−e47	−10.7 ± 2.8 #, **	−6.2 ± 0.5 ***	11	−33.6 ± 0.9 ***	8.0 ± 0.4 ###, ***	12
R1667W	+e47	−9.5 ± 0.7 **	−5.6 ± 0.2 **	21	−26.9 ± 0.8	6.8 ± 0.2	15
	−e47	−9.7 ± 3.9	−5.6 ± 1.0 ***	6	−30.0 ± 1.1	6.4 ± 0.2	7
S1799L	+e47	1.2 ± 0.5 ***	−5.8 ± 0.3 **	15	−24.5 ± 0.7	9.1 ± 0.5 **	13
	−e47	1.7 ± 2.5 ***	−5.5 ± 1.0 ***	9	−22.7 ± 0.6 ***	7.4 ± 0.3 #, **	6

Non-paired Student’s *t*-test. Asterisk (*) and number signs (#) denote significant differences between mutant and WT, and between Cav2.1+e47 and Cav2.1−e47 splice variants, respectively, with significance established as follows: ** *p* < 0.01, *** *p* < 0.001, # *p* < 0.05, ### *p* < 0.001.

**Table 2:** Inactivation kinetics and recovery from inactivation.

		R400	*n*	% Recovery at 8 s	*n*
WT	+e47	0.47 ± 0.02	22	82 ± 1	5
	−e47	0.32 ± 0.02 ###	17	85 ± 2	11
A405T	+e47	0.41 ± 0.01	28	88 ± 2 **	5
	−e47	0.35 ± 0.02 #	11	83 ± 3	8
R1359W	+e47	0.31 ± 0.03 ***	29	77 ± 4	4
	−e47	0.24 ± 0.01 ***	18	78 ± 4	12
R1667W	+e47	0.36 ± 0.02 **	44	85 ± 1	5
	−e47	0.30 ± 0.02	13	86 ± 1	9
S1799L	+e47	0.27 ± 0.02 ***	38	92 ± 1 ***	6
	−e47	0.19 ± 0.02 #, ***	13	81 ± 2 ##	10

R400 values were evaluated with depolarizing pulses to 0 mV for WT, A405T, R1359W, and R1667W variants, and to +10 mV for S1799L variants. Non-paired Student’s *t*-test. Asterisk (*) and number signs (#) denote significant differences between mutant and WT, and between Cav2.1+e47 and Cav2.1e−47 splice variants, respectively, with significance established as follows: ** *p* < 0.01, *** *p* < 0.001, # *p* < 0.05, ## *p* < 0.01, ### *p* < 0.001.

## Data Availability

The data presented in this study are available on request from the corresponding author.
